# A model for promoting breast self-examination among rural South African women in KwaZulu-Natal

**DOI:** 10.4102/hsag.v30i0.2777

**Published:** 2025-04-30

**Authors:** Nelisha Sarmah, Maureen N. Sibiya, Thandokuhle E. Khoza

**Affiliations:** 1 Department of Radiography, Faculty of Health Sciences, Durban University of Technology, Durban, South Africa; 2 Division of Research, Innovation and Engagement, Mangosuthu University of Technology, Durban, South Africa

**Keywords:** breast self-examination, model development, promotion, rural, South Africa, health education

## Abstract

**Background:**

Breast self-examination (BSE) is a safe, easy and affordable breast screening tool encouraged as an alternative screening method in low-resource settings. The late presentation of breast cancer among rural African women in South Africa is of great concern. No BSE model has been identified for promoting BSE awareness and practice among rural African women in South Africa.

**Aim:**

To describe the process involved in developing and describing the model for promoting BSE among rural African women in South Africa.

**Setting:**

Four municipal clinics in Kwa-Zulu Natal’s iLembe District Municipality.

**Methods:**

An exploratory qualitative study was conducted using semi-structured one-on-one interviews. Deductive thematic analysis was used to analyse the data. The model was then developed using Chinn and Kramer’s four-step theory generation process: concept analysis, relationship statements, model description and model evaluation.

**Results:**

A concept analysis was conducted in two phases. A central concept was identified in phase one, and in phase two, it was defined and classified. Based on the identified and defined central concepts, relationship statements were created, which added clarity and direction to the understanding of the phenomenon. The BSE model was described in terms of its purpose, structure and assumptions.

**Conclusion:**

Using the BSE model, breast cancer campaigns and programmes can be tailored to the needs of geographically disadvantaged communities in rural South Africa.

**Contribution:**

Using the BSE model, the model is proposed to improve the early detection of breast cancer.

## Introduction

Globally, breast cancer is a significant public health issue and the leading cause of death for women (Oosthuizen, Van der Merwe & Kotze 2024). Additionally, it represents 22.6% of all female cancers and 16% of cancer deaths among women in South Africa (Ayeni et al. 2023; Mashele et al. 2023; Sarmah, Sibiya & Khoza 2023). Globally, there are an estimated 2.4 million breast cancer cases recorded in 2020, representing 11.7% of all cancer cases (Mushosho et al. 2024). According to the World Health Organization (WHO), this number will rise to 19m by 2025, 22m by 2030, and 24m by 2050 (Manson & Achel 2023). In black South African women, 3-year overall survival is estimated at 59%, primarily because of late-stage diagnosis (Ayeni et al. 2023). In previous South African studies, 63.4% of breast cancer patients of African descent were diagnosed with stages III and IV of the disease (Ayeni et al. 2023; Kakudji et al. 2020; Warnich, Viljoen & Kuehnast 2020). A study of black women in South Africa found that women waited approximately 8.5 months between discovering a breast lump and developing other symptoms that caused them to seek medical attention (Lambert et al. 2020). While breast-conserving surgery is the surgical treatment of choice in high-resource settings, total mastectomy is still the most common surgical treatment today in South African public hospitals (Lambert et al. 2020). Several factors may contribute to this, including the lack of awareness of breast cancer, lack of knowledge and practice of breast self-examination (BSE) and lack of health education (Lambert et al. 2020).

South Africa faces significant challenges in the fight against breast cancer, including substantial disparities in breast cancer screening, treatment and survival as a result of ethnic and socioeconomic factors (Dlamini, Molefi & Khanyile 2024). In addition, there are racial disparities in screening, incidence and survival, leading to delayed diagnosis among black women and highlighting healthcare inequities (Dlamini et al. 2024). However, obstacles within the healthcare system impede progress (Dlamini et al. 2024), which underscores the urgency of enhancing breast cancer screening while mitigating treatment delays. The importance of robust screening programmes, particularly those targeting marginalised communities, cannot be overstated. The regular use of BSE, along with breast cancer awareness, is one of the strategies for achieving an early diagnosis of breast cancer among rural South African women. Therefore, a model for promoting BSE knowledge and practice is suggested to improve early detection of breast cancer among rural South African women.

### Problem statement

Screening by mammography is inaccessible to millions of women living in developing countries, including South Africa, as it is technologically and financially difficult to implement and maintain (Dlamini et al. 2024). There are insufficient resources in South Africa to implement and sustain a national screening programme (Warnich et al. 2020). For this reason, the South African Department of Health (DoH) recommends BSE as an early detection method for women in low-resource settings. Although BSE is safe, easily accessible and highly recommended for women in low-resource settings, its uptake is poor in LMICs like South Africa (Udoh et al. 2020).

As part of a national campaign to raise awareness of breast cancer, the South African DoH has designated October as ‘Breast Cancer Awareness Month’. However, most campaigns are conducted in urban areas, and this message may not reach women in geographically disadvantaged communities. In addition, the Health Professions Council of South Africa (HPCSA) has ruled that radiographers can perform mammograms if they possess a postgraduate qualification (HPCSA 2022). There are, however, no guidelines for training and disseminating BSE knowledge and practices among health professionals and the public. Moreover, PinkDrive, a South African breast cancer public benefit organisation, is driving the country’s first mobile mammography and education unit which emphasises the importance of early detection (PinkDrive 2022). However, because of financial constraints and a lack of resources, this organisation cannot access every rural area in South Africa to promote breast cancer screening. As part of its breast cancer screening initiatives, the Cancer Association of South Africa (CANSA) supports the availability of information online and in local communities (CANSA 2020). However, women in rural communities often do not have access to these initiatives.

Currently, there are no models designed to promote BSE among rural South African women. The socioeconomic and cultural differences between rural South African women and those in other countries precluded the application of BSE models developed in other countries. It was, therefore, necessary to develop a model specifically tailored for rural South African women to promote BSE knowledge and practice.

## Research methods and design

### Research purpose

This study aimed to develop a model for promoting BSE knowledge and practice among rural South African women.

### Definition of central concepts

#### Model

A model is a schematic representation of reality or one’s view of the world, constructed to make predictions about the world and/or gain a deeper insight (Wunsch 1994). Models are intermediate between theory and data. The use of data allows theories and models to be confirmed or falsified (Wunsch 1994).

#### Rural

Sparsely populated areas based on farming and natural resources, including villages and small towns, are devoted to traditional settlements and agriculture (Department of Treasury 2011). These areas include large settlements in the former homelands and depend on migratory labour for their survival as well as government social grants (Department of Treasury 2011).

#### Breast self-examination

Breast self-examination involves visualising and palpating the breast to determine lumps, shape, texture, size and contours (Johnson 2019).

### Development of a model to promote breast self-examination among rural South African women

In this study, the model was developed according to Chinn and Kramer’s four-step theory generation process (Chinn & Kramer 2018). There are four steps involved in this process: concept analysis, relationship statements, model description and model evaluation.

### Step 1: Concept analysis

Concept analysis refers to the process of determining a concept’s semantic structure (Walker & Avant 2019). The concept analysis was conducted in two phases. In phase one, the central concept was identified and in phase two, it was defined and classified.

### Phase one: Concept identification

**FIGURE 1:**
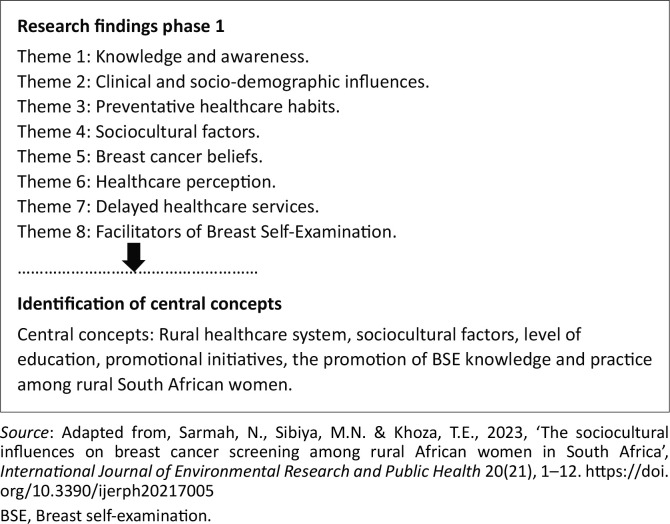
Identification of the central concepts.

These central concepts were derived from the findings in phase one published in Sarmah et al. (2023). Figure 1 illustrates the central concepts arising from the factors influencing the practice of BSE among rural South African women. Using semi-structured one-on-one interviews, the researcher developed eight themes and identified the central concepts by deductive thematic analysis. The study identified several concepts, including the rural healthcare system, sociocultural factors, education and promotional initiatives. There was a significant influence of these concepts on rural African women’s knowledge and practice of BSE in South Africa. Thus, the central concept of this study was the promotion of BSE knowledge and practice among rural South African women. Educating rural South African women about BSE will contribute to the early detection of breast cancer. The research design and findings of the first phase are summarised below.

#### Research design

The model was developed using a deductive strategy and an exploratory qualitative research method.

#### Population and sample

The study population consisted of rural South African women attending one of the municipal clinics, 20 years and older, living within the iLembe District Municipality. The participants were selected using a purposive sampling method if they met the inclusion criteria. As a criterion for participation, participants had to be South Africans of African descent aged 20–60, living in rural areas of the iLembe district in the KwaZulu-Natal (KZN) province and consenting to participate in the study. Data saturation was used to determine the sample size (Mwita 2022). A total of 22 rural South African women participated in the study.

#### Data generation procedure

The data were collected between August 2022 and October 2022. The data for this study were collected from four municipal clinics located in the iLembe district at Maphumulo, Ndwedwe, Mandeni and KwaDukuza. As the most convenient facility for most rural women in attendance, these clinics were used to interview participants. The data were obtained through semi-structured interviews using open-ended questions (Appendix 1) and probing when necessary. To ensure confidentiality, interviews were conducted in a designated area within the clinic. The use of standardised interview guides assured consistency, stability and repeatability of participants’ perspectives. This also ensured the credibility of the interviews. The duration of each interview ranged between 30 min and 40 min. In total, 22 interviews were conducted. Saturation of data was achieved with 18 participants, but four additional interviews were conducted to ensure that data saturation was reached. With the permission of each participant, the interviews were conducted in English, audio recorded and later transcribed verbatim.

#### Data analysis

The researcher transcribed the interviews verbatim, replacing participant names with codes to ensure confidentiality. Data analysis was conducted using deductive thematic analysis. In thematic analysis, patterns (themes) are identified, analysed and reported within the data (Guest, Macqueen & Namey 2012). The purpose was to provide a comprehensive overview and description of the data and interpret various aspects of the research study.

#### Measures to ensure trustworthiness

Trustworthiness can be defined as the degree of confidence researchers have in their data. Trustworthiness was measured using the criteria of credibility, transferability, dependability and confirmability (Enworo 2023; Lincoln & Guba 1985). Each of these criteria was applied to this study. As a result of the extensive engagement with participants, the study gained a high degree of credibility. A record of raw data for each interview was maintained to ensure reliability. A complete audit trail describing data collection, analysis and interpretation was necessary to establish confirmability. The validity and authenticity of the research were confirmed through a detailed description of both the research setting and methods. Therefore, future research could be based on this study’s findings.

### Ethical considerations

Ethical clearance was obtained from the Institutional Research Ethics Committee (IREC 157/22) at the Durban University of Technology for this study. The gatekeeper’s permission was obtained from the KZN DoH and the iLembe District Manager. A consent form was signed by all participants to participate in this study and to permit the researcher to record the interviews. Several ethical principles were considered (Hammersley & Traianou 2012), including respect for autonomy, non-maleficence, beneficence and justice. As part of respecting autonomy, the participants were allowed to make an informed decision before signing an informed consent form. The confidentiality of the participants was maintained throughout the research by not disclosing their names. Participants in the study participated voluntarily and were free to withdraw at any time during the study. All participants were assured of their privacy during the interview process. To ensure non-maleficence, a safe and comfortable environment was provided during the interview to ensure no physical harm was caused to the participants. The researcher ensured beneficence by being sensitive during the interviews and avoiding harm and discomfort to the participants. By selecting participants (African women) who are least represented in literature, living in rural South Africa (geographically remote areas) for this study, the researcher adhered to the principle of justice (Ntshingila et al. 2021).

## Results

Based on the findings of phase one, knowledge and awareness, clinical and socio-demographic influences, preventative healthcare habits, sociocultural factors, breast cancer beliefs, healthcare perception, delayed healthcare services and facilitators of BSE contributed to the low uptake of BSE among rural South African women. This will be addressed in greater detail under the heading ‘Concept analysis’ in the ‘Findings and discussions’ section.

### Phase two: Definition and classification of the central concept

According to Chinn and Kramer (2018), concepts can be derived from experiences, clinical practice, research, literature knowledge and formal conceptualisation processes. The findings of the interview are similar to those identified in phase one. Thus, socio-demographic influences, preventative healthcare habits, sociocultural factors, breast cancer beliefs, healthcare perception and delayed healthcare services, coupled with a lack of awareness and practice of BSE, formed the basis for the model (Udoh et al. 2020).

### Step 2: Relationship statement

Chinn and Kramer (2012) define statements as relational or non-relational. A relational statement implies some connection between two or more concepts while confirming causality or association. In associational statements, similar concepts and casual statements are grouped to determine the cause-and-effect relationship (Walker & Avant 2019). In a non-relational statement, concepts are affirmed and model meaning is clarified (Chinn & Kramer 2012). It was necessary to place the identified central concepts into relationship statements to develop a model for promoting BSE knowledge and practice. The relationships were constructed using the essential and related attributes of BSE promotion. Regarding promoting BSE, the following attributes were identified: the rural healthcare system, sociocultural factors, education levels and promotional activities. The attributes resulted in the definition of the central concept of ‘BSE promotion’, the identification of the factors influencing BSE practice, and the inclusion of key stakeholders during promotional campaigns will result in increased BSE awareness and practice among rural African women. By identifying these factors, targeted campaigns will be designed for rural African women in South Africa that take into account their socioeconomic and cultural backgrounds.

### Step 3: Model description

The model is based on Chinn and Kramer’s components of a theory. These components include purpose, concept definition, relationship statements, the structure of the model and assumptions. The South African DoH may utilise the BSE promotional model to promote BSE screening as an alternative to mammography screening which is generally inaccessible to rural women.

### Step 4: Model evaluation

Chinn and Kramer recommend critical reflection to determine how well a model meets its objectives. This study did not evaluate the model because it was not one of the objectives of this study. This serves as a limitation to the study.

## Findings and discussions

The results are given according to the steps in the model development as prescribed by Chinn and Kramer.

### Step 1: Concept analysis

According to this study, 8 main themes and 20 sub-themes emerged. The findings of this study and the literature review served as the basis for the classification of concepts and development of the model. An overview of the findings that formed the basis for the development of the model can be found in Table 1.

**TABLE 1:** Factors influencing the knowledge and practice of breast self-examination.

Theme	Sub-theme	Qualitative findings
1. Knowledge and awareness	1.1 Breast cancer misconception 1.2 Lack of breast self-examination knowledge	‘It’s something that is not curable.’ (P2, high school, unemployed) ‘We don’t know anything about breast exams.’ (P10, high school, part-time employed)
2. Clinical and socio-demographic influences	2.1 The influence of education on BSE knowledge and practices	‘The educated ones, they will understand what is breast cancer and others who don’t have knowledge about it, they think someone who got breast cancer is bewitched.’ (P18, high school, part-time employed)
3. Preventative healthcare habits	3.1 Clinics and hospitals 3.2 Self-treatment practices for medical symptoms 3.3 Complementary practices	‘I will see a doctor.’ (P4, high school, unemployed) ‘I will just make what the people tell me I must make and drink, like ginger, fresh turmeric roots.’ (P12, high school, unemployed) ‘I use traditional herbs.’ (P2, high school, unemployed)
4. Sociocultural factors	4.1 Fear 4.2 African cultural stigmatisation 4.3 Social support for African women 4.4 Positive attitude on BSE practices	‘This illness is like we scared to have it because we think that it’s not curable, and you’re going to die.’ (P3, high school, unemployed) ‘They will mistreat her. They will see her as a different person like she’s not qualified to be a woman.’ (P2, high school, unemployed) ‘I want to learn more. I want to know more.’ (P3, high school, unemployed)
5. Breast cancer beliefs	5.1 Traditional beliefs 5.2 Healthcare beliefs	‘Mostly, they think of witchcraft. They think maybe you are cursed.’ (P20, high school, unemployed) ‘It is a health problem because if it was a spiritual one, I would consult a traditional healer.’ (P5, tertiary education, part-time employed)		
6. Healthcare perception	6.1 Healthcare perception	‘If we have someone who knows about it, like for instance, I know about the examination. So, if I teach my family obviously, they will practice about it.’ (P1, high school, full-time employed)		
7. Delayed healthcare services	7.1 Health practitioner’s conduct 7.2 Educative material 7.3 Resources and infrastructure 7.4 Rural transport services 7.5 Individual healthcare affordability	‘…. from my side I had a problem before where I had to go to the nurse, and she had to scream which room I must go to.’ (P10, high school, part-time employed) ‘From my understanding, they only give it if it’s breast cancer week. If not, there’s nothing.’ (P5, tertiary education, part-time employed) ‘Transport. They don’t give to me on time because where I live it’s in a rural area so there is less transport.’ (P2, high school, unemployed) ‘The problem I have when I need medical is a money. Sometimes I’m sick, I don’t have the money.’ (P16, high school, part-time employed)		
8. Facilitators of Breast Self-Examination	8.1 Community engagement 8.2 Health Education	‘Organising programmes or campaigns, breast cancer campaigns, get women to get together and educate them.’ (P18, high school, part-time employed) ‘I think that they must come and educate the people from the community.’ (P14, high school, full-time employed)

Source: Adapted from, Sarmah, N., Sibiya, M.N. & Khoza, T.E., 2023, ‘The sociocultural influences on breast cancer screening among rural African women in South Africa’, *International Journal of Environmental Research and Public Health* 20(21), 1–12. https://doi.org/10.3390/ijerph20217005

BSE, Breast self-examination.

Many participants in this study expressed concerns about the quality of the rural healthcare system’s infrastructure and services. Studies have reported that rural women in South Africa are more likely to present late with breast cancer (Bhuiyan et al. 2022; Nwagu et al. 2021). This was because of a lack of medical professionals, screening technologies, resources, distance to the nearest health facility, transportation issues and treatment facilities in many hospitals. As a result, healthcare services were delayed, and women did not follow through with mammogram referrals (Akuoko et al. 2017).

The findings of this study indicate that sociocultural factors (affect, habit, belief and norm) significantly influenced participants’ knowledge and practice of BSE. Several participants expressed confidence in traditional healing practices, traditional healers and self-treatment alternatives if they felt pain, lump or changes in their breast. Several studies have shown that cultural, religious and mental beliefs and habits contribute to delays in the diagnosis and treatment of cancer in women, including beliefs in witchcraft and evil spirits (Elewonibi & BeLue 2019; Nwagu et al. 2021; Ramathuba, Ratshirumbi & Mashamba 2015).

Several participants expressed concern and fear regarding the stigmatisation of women suffering from breast cancer. A study conducted in Ethiopia and Uganda found that public stigmatisation associated with breast cancer led to internalised stigmatisation, which in turn delayed care engagement (Meacham et al. 2016). Participants in this study also believed that women who have support from their family, friends, and community are more likely to practice breast cancer screening.

**FIGURE 2:**
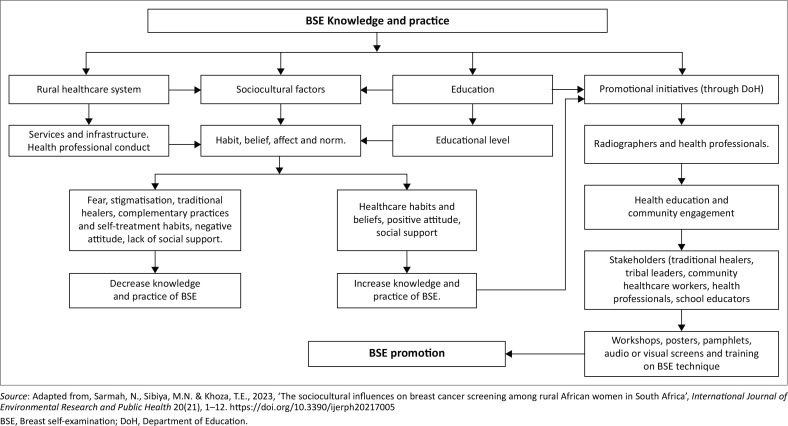
The breast self-examination model.

A study in rural Uganda found that low levels of education were associated with a lack of knowledge and practice of BSE, especially in populations characterised as economically disadvantaged and uneducated (Joyce, Ssenyongaa & Iramiotb 2020). This study also found that women with a lower education level had several misconceptions regarding breast cancer compared to women with a tertiary education. Many participants indicated that breast cancer misconceptions and the reluctance to practice BSE among rural South African women were because of a lack of awareness, educational materials in PHC facilities and promotional efforts. Azemfac et al. (2019) recommend that a model for disseminating breast cancer knowledge in low-resource settings be developed. In response to the findings of this study, coupled with an extensive literature review and the need to promote BSE among rural South African women, the BSE promotional model was developed, as shown in Figure 2.

### Step 2: Relationship statements

The identified and defined central concepts were placed into relationship statements (Chinn & Kramer 2012). Relationship statements help to give the concepts more clarity and add direction to the understanding of the phenomenon. In this study, the relationship statements were formulated as follows:

BSE knowledge and practice improve when:

- The South African rural healthcare system is improved through its services, infrastructure and health professionals.
- An increase in education will lead to an improvement in sociocultural behaviour patterns and promotional initiatives.
- Promotional breast initiatives will be improved through the inclusion and collaboration with influential community stakeholders.

There is a lack of knowledge and practice of BSE when:

- Poor rural healthcare system increases complementary practices and habits.
- The lack of education leads to several misconceptions regarding breast cancer practices.
- Key stakeholders are excluded during promotional initiatives.
- There is a lack of social support.
- The traditional wellness approach (habit, belief, norm) is preferred to conventional healthcare.

### Step 3: Model description

The BSE promotional model is described in terms of purpose, structure and assumptions (Chinn & Kramer 2012).

#### Purpose of the model

There should be consideration of who will implement the model and under what conditions or circumstances (Chinn & Kramer 2012). The goal is to use this model as a framework for tailoring breast cancer awareness campaigns in rural areas of South Africa.

#### Structure of the model

A schematic representation is shown in Figure 2 of the BSE promotional model, which focuses on identifying BSE facilitators, key stakeholders, community engagement and health education in rural South African communities.

#### Assumptions of the model

Assumptions are defined by Chinn and Kramer (2018) as underlying, given that they are presumed true. South African rural women’s knowledge and practice of BSE are influenced by four factors, namely the rural healthcare system, sociocultural factors, education level and promotional initiatives. The South African rural healthcare systems such as infrastructure and services offered have an impact on BSE knowledge and practice. Breast cancer awareness initiatives can be enhanced by optimising and improving resources, infrastructure and services.

The model also incorporates sociocultural factors, which suggest that rural South African women’s knowledge and practice of BSE are influenced by affect, habit, belief and norms. By understanding these factors, awareness programmes regarding BSE can be tailored and considerate to the sociocultural factors affecting a particular group of women. There were several sociocultural factors identified, including misconceptions, stigmatisation, fear, beliefs and habits. Additionally, sociocultural factors will aid in the identification of influential key stakeholders who can be used to promote BSE within communities. Among the other factors identified were social support, attitude and the use of complementary practices.

It was also noted that women’s educational level was a contributing factor that influences the knowledge and practice of BSE. It is suggested that women with higher levels of education are more likely to be familiar with BSE and more likely to practice it. Promotional initiatives were also identified as a significant factor influencing BSE knowledge and practice. Based on this study, rural South African communities lack educational material regarding breast cancer screening. Therefore, health education and community engagement through workshops, posters, pamphlets, audio-visual screens and training on BSE techniques will be necessary to teach key stakeholders BSE techniques and frequency, with the approval of the DoH and the use of highly skilled healthcare professionals, such as mammographers. As part of this model, tribal leaders, traditional healers, school educators, community healthcare workers and health professionals were identified as key stakeholders. These key stakeholders are encouraged to disseminate BSE knowledge and practice within their respective communities. In this way, more women in geographically disadvantaged communities will practice BSE and present to the nearest PHC facility for breast abnormalities, symptoms and concerns.

### Step 4: Model evaluation

The model has not been evaluated yet and will be conducted as postdoctoral study. Nevertheless, a critical reflection was conducted using Chinn and Kramer’s (2012) five elements of critical reflection, namely clarity, simplicity, generality, accessibility and importance.

#### Clarity

In this model, core concepts were used to achieve semantic clarity, and no novel concepts were introduced. In this model, the concepts were consistent and the structure was clear.

#### Simplicity

In this model, the structural components and their relationships are indicated and explained (Chinn & Kramer 2012). A clear description of the factors that increase or decrease knowledge and practice of BSE is provided, along with steps that can be utilised to promote BSE knowledge and practice.

#### Generality

Generality refers to the breadth and scope of the model’s application (Chinn & Kramer 2012). While the model was designed to promote BSE knowledge and practice among rural South African women, it can also serve as a guideline for developing other promotional campaigns in the communities, such as HIV campaigns. Additionally, it helps identify key role players within rural communities who can support the early detection of cancer or other diseases. Furthermore, the barriers that affect BSE knowledge and practice can also be used to identify disparities in rural South African communities.

#### Accessibility

Accessibility measures the extent to which indicators for concepts can be identified and the extent to which their purpose can be achieved (Chinn & Kramer 2012). The model will be presented to the DoH for review and approval. It will also be presented to health professionals at PHC facilities to promote BSE knowledge and practice among rural South African women. This will be performed with the cooperation and collaboration of key stakeholders.

#### Importance

The importance of a model is determined by its practical value or significance (Chinn & Kramer 2012). Through the knowledge and practice of BSE, the model aims to improve the early detection of breast cancer among rural South African women. It also highlights the barriers to breast cancer screening, which exist in South African rural communities that require urgent attention as well as the implementation of health education and community engagement strategies.

## Limitations

The BSE model was not evaluated and thus serves as a limitation of the study. It should be noted that the study was conducted exclusively in rural South Africa. Changes to the model may be necessary if replicated in other regions as a result of context differences.

## Recommendations

It is recommended that future research examines the role of key stakeholders in promoting BSE in rural South African communities. Furthermore, future research should include the development of an evaluation tool to test the model in rural communities in South Africa. Governing bodies should use the model as a guide to ensure that breast cancer awareness campaigns are tailored to reach women in geographically disadvantaged communities and to ensure that best practices and health education are provided to all women. Further, a collaborative partnership between health professionals and community leaders must be strengthened to promote knowledge and practice of BSE in rural South African communities.

## Conclusion

There was an urgent need to develop a model to aid in the promotion of BSE among rural South African women who were presenting with advanced-stage breast cancer. Through the collaboration and cooperation of influential key stakeholders identified in this model, the BSE promotional model intends to improve knowledge and practice of BSE among rural South African women. It also aims to provide an alternative decision-making process for addressing the challenges facing breast cancer screening in rural South African communities.
